# Information Exchange Design Patterns for Robot Swarm Foraging and Their Application in Robot Control Algorithms

**DOI:** 10.3389/frobt.2018.00047

**Published:** 2018-06-07

**Authors:** Lenka Pitonakova, Richard Crowder, Seth Bullock

**Affiliations:** ^1^Department of Computer Science, University of Bristol, Bristol, United Kingdom; ^2^Department of Electronics and Computer Science, University of Southampton, Southampton, United Kingdom

**Keywords:** swarm robotics, design patterns, foraging, communication, information, control algorithm, bee-inspired, ant-inspired

## Abstract

In swarm robotics, a design pattern provides high-level guidelines for the implementation of a particular robot behaviour and describes its impact on swarm performance. In this paper, we explore information exchange design patterns for robot swarm foraging. First, a method for the specification of design patterns for robot swarms is proposed that builds on previous work in this field and emphasises modular behaviour design, as well as information-centric micro-macro link analysis. Next, design pattern application rules that can facilitate the pattern usage in robot control algorithms are given. A catalogue of six design patterns is then presented. The patterns are derived from an extensive list of experiments reported in the swarm robotics literature, demonstrating the capability of the proposed method to identify distinguishing features of robot behaviour and their impact on swarm performance in a wide range of swarm implementations and experimental scenarios. Each pattern features a detailed description of robot behaviour and its associated parameters, facilitated by the usage of a multi-agent modeling language, BDRML, and an account of feedback loops and forces that affect the pattern’s applicability. Scenarios in which the pattern has been used are described. The consequences of each design pattern on overall swarm performance are characterised within the Information-Cost-Reward framework, that makes it possible to formally relate the way in which robots acquire, share and utilise information. Finally, the patterns are validated by demonstrating how they improved the performance of foraging e-puck swarms and how they could guide algorithm design in other scenarios.

## 1. Introduction

Multi-robot engineering is challenging, as it often requires a “bottom-up” approach to behavioural design (Trianni et al., 2011; Parunak and Brueckner, 2015). The emergent macro behaviour of the swarm is specified and evaluated, but it is the micro behaviour of individual robots that needs to be programmed. Furthermore, the relationship between these two levels is often somewhat opaque. There are a number of possible approaches when it comes to choosing algorithms for robot swarms, including, for instance, selecting an arbitrary algorithm either inspired by nature or by previous work in robotics (e.g., Sugawara and Watanabe, 2002; Lemmens et al., 2008; Gutiérrez et al., 2010; Fujisawa et al., 2014; Pitonakova et al., 2016a), using artificial evolution to select robot behaviours (e.g., Sperati et al., 2011; Ferrante et al., 2015)and applying design patterns.

In software engineering, a design pattern represents implementation-generic guidelines for a part of a system’s behaviour, usually created as an abstraction from a previously implemented algorithm, that can be applied to a class of similar problems (Gamma et al., 1994; Do et al., 2003). The potential of design patterns to facilitate reliable and efficient creation of algorithms has already been recognised in the swarm robotics literature (e.g., Nagpal, 2004; Parunak and Brueckner, 2004; Serugendo et al., 2006; Gardelli et al., 2007; Winfield, 2009b; Reina et al., 2015), although a number of challenges remain partially unsolved. The first challenge in design pattern creation is establishing a common language for describing them (Graves and Czarnecki, 2000). Secondly, identifying a framework within which the effect of design patterns on collective swarm performance can be analysed is important (Gardelli et al., 2007; Reina et al., 2014). Thirdly, swarm design patterns should be created based on an extensive set of experiments and attention should be paid to their generality and reusability (Fernandez-Marquez et al., 2013).

These challenges are addressed here in the following ways. In order to address the first two problems related to design pattern specification, a new method for describing and combining design patterns for robot swarm foraging is proposed, utilising information-centric approaches that include the Information-Cost-Reward (ICR) framework for swarm behaviour analysis (Pitonakova et al., 2018) and a formal modelling language for multi-agent systems, BDRML (Pitonakova et al., 2017). The main objectives of a design pattern, as defined here, are 1. to specify a self-contained module of robot behaviour in terms of robot actions and data dependencies using BDRML; 2. to identify the properties of this module, such as control parameters and their effect on the overall swarm performance within the ICR framework; and 3. to advise on the suitability of using the module in the context of other robot behaviours and swarm task parameters. The emphasis on pattern modularity is an important one. As will be demonstrated here, by creating patterns that only describe a particular aspect of a robot control algorithm (e.g., a location at which robots exchange information), it is possible to study and clearly describe the design pattern’s properties and consequences, as well as to create a range of robot control algorithms by considering the relationships between multiple design patterns and by applying well-specified Application Rules to their BDRML representations.

This work focuses on design patterns for information exchange during multi-robot foraging, where robots need to search an unknown environment for *worksites* and either perform work on them or transport goods from the worksites into a predefined location. The robots may or may not exchange information about the worksites. Foraging was selected because it is often used as a paradigm for a wide range of real-world robot collective tasks such as collection of resources, search and rescue operations, environment cleanup, customer servicing, etc. (e.g., Gutiérrez et al., 2010; Jevtic et al., 2012; Lee et al., 2013; Ducatelle et al., 2014). Information processing and exchange have previously been emphasised as important elements of any swarm behaviour (e.g., Trianni et al., 2011; Wang et al., 2012; Fernandez-Marquez et al., 2013; Miller et al., 2014; Reina et al., 2015), and are therefore the main focus of the patterns presented here. The ways in which similar design patterns for other collective swarm tasks could be created are discussed in Section 6.

In order to consider a sufficient breadth of research work, a catalogue of six design patterns is presented, that is based on an extensive literature review, including our previous swarm analysis work. The design pattern creation method proposed here is applied to reason about and to organise behaviours repeatedly implemented in the literature, demonstrating its ability to identify distinguishing features of robot behaviour and their impact on swarm performance in a wide range of swarm implementations and experimental scenarios. Finally, in order to validate the design patterns and to demonstrate their applicability, examples of real-world and hypothetical scenarios where the patterns have or could be combined into robot control algorithms in order to improve swarm performance, given the mission requirements and robot hardware constraints, are provided.

The rest of the paper is organised as follows. In the next section, the background related to design patterns, the ICR framework and BDRML is provided. The method for creating, representing and applying design patterns is introduced in Section 3. The Design Pattern Catalogue is provided in Section 4 and examples of design pattern applications are included in Section 5. A more general discussion of the methods presented here, of other robot algorithm creation methods, as well as of how design patterns could complement automated robot algorithm design, such as on-line learning and artificial evolution, is provided in Section 6.

## 2. Background

### 2.1. Design Patterns

A design pattern offers a flexible high-level solution to a class of problems, that a programmer can implement in a particular, context-specific way (Do et al., 2003). Good patterns that use a common unambiguous language can decrease system design time, as well as improve communication between engineers (Brazier et al., 2002; Gardelli et al., 2007). In object-oriented software engineering, design patterns define what roles object classes should have and how they should interact (Gamma et al., 1994). In agent-based software engineering, design patterns can define roles and interactions of agents, as well as the role of the environment (e.g., Aridor and Lange, 1998; Do et al., 2003). In swarm robotics, a design pattern usually defines a part of a robot control algorithm that is responsible for a specific robot behaviour (e.g., Nagpal, 2004; Gardelli et al., 2007; Fernandez-Marquez et al., 2013). Another class of design patterns for swarm robotics includes those composed of lower-level patterns. For example a “Gradient” pattern can be composed from a “Spreading” and an “Aggregation” patterns (Fernandez-Marquez et al., 2013).

An important property of design patterns is their modularity (Mikkonen, 1998). A single pattern usually defines a solution to a specific problem, e.g., in the case of swarm robotics, how to navigate the environment, how to manage data, etc. Multiple design patterns are then combined within a single program and the programmer decides how to implement them together, given a specific application and hardware available (Hernández et al., 2013; Fernandez-Marquez et al., 2013).

The first object-oriented design patterns included several properties, including (Gamma et al., 1994):

- The pattern’s *name* and, if applicable, *aliases*
- The *intent*, i.e, the main goal of the pattern and *motivation*, i.e., the reasons why the pattern should be applied
- *Applicability*, i.e., a description of the circumstances under which the pattern should be applied
- The class *structure*, a detailed description of the *participants* identified within the structure, as well as of their *collaborations*
- A list of *consequences* on the overall software, including the advantages and disadvantages of using the pattern
- Guidance on *implementation*, including *sample skeleton code*
- A list of *known uses* of the pattern in real-world applications
- A list of *related patterns*

The above specification method was also followed in early multi-agent system patterns (Aridor and Lange, 1998). However, in later multi-agent work, the agent behaviour specification became more detailed and included the *social dimension*, that identified relevant agents, the *intention dimension*, that identified the services provided by agents, the *structural dimension*, that described how the services worked, the *communication dimension*, that modeled temporal exchange of events between agents and the *dynamical dimensions*, that described synchronisation mechanisms of the pattern (Do et al., 2003). On the other hand, other properties of design patterns, such as their applicability and their effect on the system as a whole were less prominent. Similarly, in early multi-robot design pattern work (Nagpal, 2004), the patterns only included a description of how they worked, but did not describe their context or consequences.

Later multi-robot design patterns reverted to the detailed format that was originally used in object-oriented software (Gamma et al., 1994), but also featured additional information due to the complex nature of multi-robot systems, including (Gardelli et al., 2007; Fernandez-Marquez et al., 2013):

- The *problem description*, that replaced intent and motivation and that either described a particular pathological collective behaviour that a pattern was created to prevent, or a particular behaviour that the multi-robot system could achieve
- The *forces*, i.e., the way in which the pattern’s parameters affected its effectiveness
- The description of a solution, including a list of *entities* and their *dynamics*, the *feedback loops* involved, an *example* that graphically described a possible implementation of the pattern, including guidance on how such implementation could be realised.
- The *inspiration* behind the pattern in the form of its equivalents found it nature.

### 2.2. Graphical Representation of Design Patterns

An important part of a design pattern is a description of the behaviours that the pattern represents, facilitated by a well-specified modelling language with an unambiguous syntax and semantics (Harel and Rumpe, 2004), and preferably, with graphical elements (Graves and Czarnecki, 2000). In object-oriented software engineering, UML class diagrams^[1]^ are commonly used to graphically represent object classes and their relationships. Multi-agent software patterns additionally make use of state charts (Castello et al., 2016) and sequence charts (Do et al., 2003).

**Figure 1:**
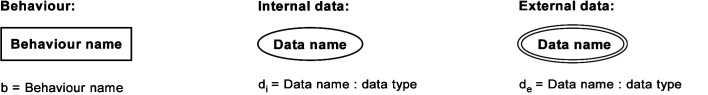
Graphical and textual representations of BDRML primitives. Reproduced from (Pitonakova et al., 2017) with permission of the copyright holder, IEEE.

In this paper, the *Behaviour-Data Relations Modelling Language* (BDRML) (Pitonakova et al., 2017), that was specifically designed for multi-agent systems, is used. In BDRML, agent behaviours and data structures, as well as their relationships, are represented explicitly, allowing the language to represent multi-agent control algorithms, as well the way in which agents interact with each other and with their environment. This makes BDRML a suitable choice for representing design patterns for robot swarms, where information processing and exchange play a pivotal role (Trianni et al., 2011; Wang et al., 2012; Fernandez-Marquez et al., 2013; Miller et al., 2014; Reina et al., 2015). BDRML defines three types of *primitive* (Figure 1):

- ***Behaviour***, i.e., a set of processes that deal with a particular situation that a robot may find itself in, for example, “Scout”
- ***Internal data structure***, i.e., information that is stored in a robot’s memory
- ***External data structure***, i.e., information that is stored in a non-robot entity, i.e., in the robot’s environment

**Figure 2:**
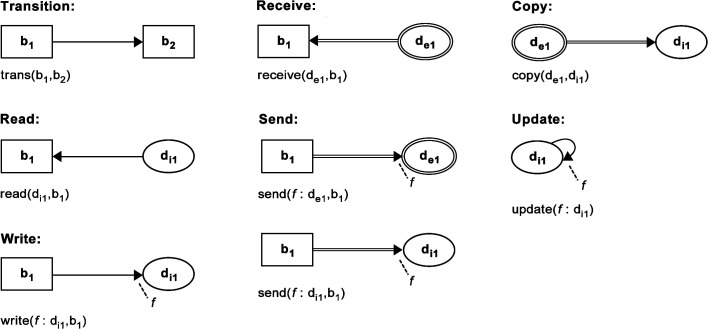
Graphical and textual representations of BDRML relations and operations. Reproduced from Pitonakova et al. (2017) with permission of the copyright holder, IEEE.

BDRML primitives can be linked by the following relations (Figure 2):

- ***Transition:*** The robot transitions from one behaviour to another
- ***Read/Write:****Internal* data is used/stored by the robot when it is engaged in a particular behaviour
- ***Receive/Send:****External* data is used/stored by the robot. In the case of the *Send* relation, a robot may also send the data to another robot that stores it in its own *internal* data structure
- ***Copy:*** Information is copied from one data structure to another
- ***Update:*** The value of a data structure is updated from that in the previous time step by a subroutine not visualised in the BDRML diagram (for example, a pheromone level may “spontaneously” decrease over time).

The *write* and *send* relations can optionally define the new data structure value or a function that updates the value, indicated by a dashed line extending from the end of the relation arrow in a graphical description, and written before a colon proceeding the data structure name in a textual description. The *update* relation must always specify the new value or the value update function.

**Figure 3:**
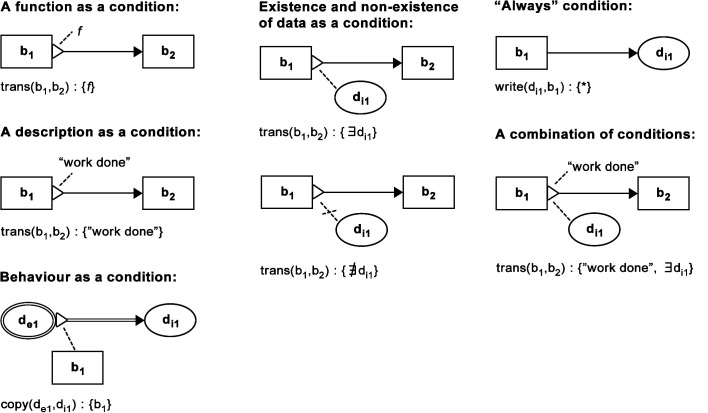
Graphical and textual representations of BDRML conditions. Reproduced from Pitonakova et al. (2017) with permission of the copyright holder, IEEE.

Finally, each relation or operation occurs under a specific set of *conditions* (Figure 3). A condition is graphically represented as an annotated triangle at the beginning of a relation arrow. In a textual representation, a condition set follows a relation signature and is separated from it by a colon. Unless otherwise specified, the *or* logical operator is used when multiple conditions affect a single relation.

**Figure 4:**
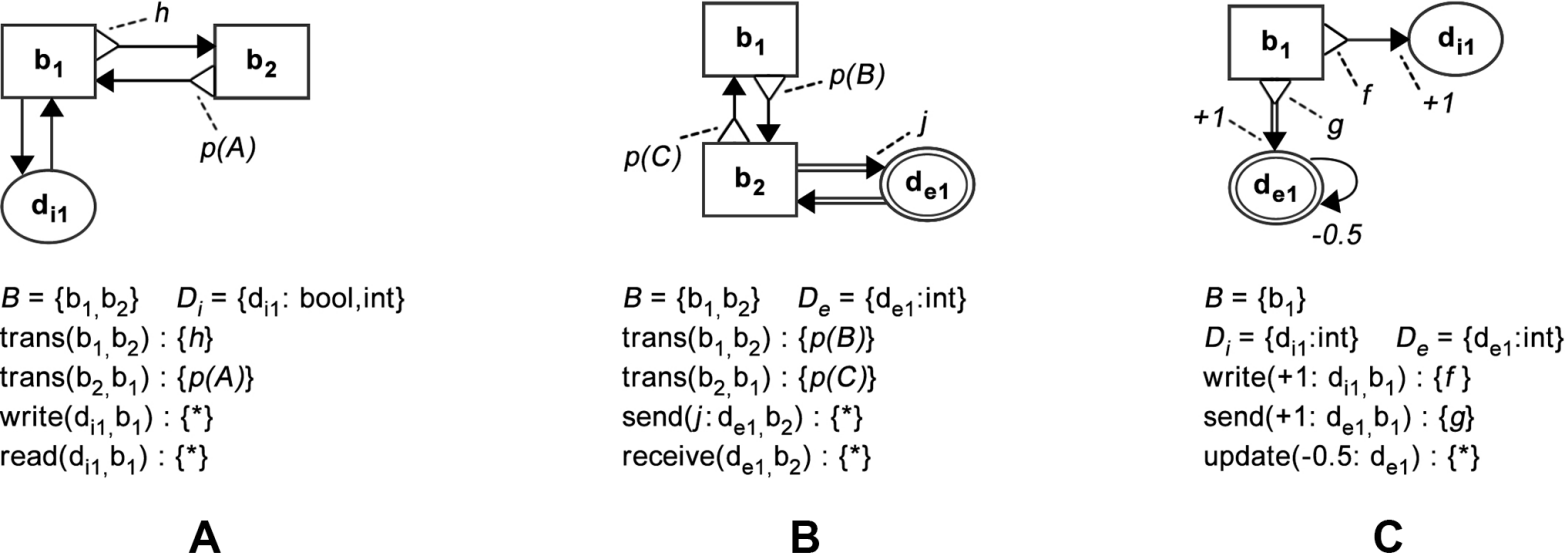
BDRML examples of design patterns. **(A)** A pattern consisting of behaviours $b_{1}$ and $b_{2}$ and of an internal data structure of boolean or integer type, $d_{i1}$. A robot transitions from $b_{1}$ to $b_{2}$ when a boolean function h returns *true*. The robot transitions back from $b_{2}$ to $b_{1}$ with a probability p(A). While performing $b_{1}$, the robot writes into and reads from $d_{i1}$. **(B)** A pattern consisting of behaviours $b_{1}$ and $b_{2}$ and an external data structure $d_{e1}$. The robot transitions probabilistically between $b_{1}$ and $b_{2}$ and updates the value of $d_{e1}$ according to the function j. **(C) **A pattern consisting of behaviour $b_{1}$, an internal data structure $d_{i1}$ and an external data structure $d_{e1}$. While in $b_{1}$, the robot may update the value of $d_{i1}$ by +1, provided that the boolean function f returns *true*. Similarly, the robot may update $d_{e1}$ when g returns *true*. Additionally, the value of $d_{e1}$ is updated by −0.5 at each time step.

A BDRML representation of a design pattern includes a set of behaviours, B, a set of internal, $D_{i}$, and external, $D_{e}$, data structures and a list of conditional relations. Figure 4 shows BDRML representations of three example design patterns.

### 2.3. The Information-Cost-Reward Framework

The Information-Cost-Reward (ICR) framework (Pitonakova et al., 2018) formally relates the way in which robots obtain and share information (e.g., about worksites to forage from) to the swarm’s ability to use that information in order to obtain reward efficiently, given a particular swarm task and environment. Using this framework can address concerns regarding generality and reusability of patterns (Fernandez-Marquez et al., 2013), as well as those regarding describing the effect of patterns on collective swarm performance (Gardelli et al., 2007; Reina et al., 2014).

The framework identifies various metrics and formally relates them to the amount of reward that a swarm is able to obtain at a given point in time. *Scouting efficiency* and *information gain rate* characterise how well robots are able to obtain new information about worksite locations and share it amongst themselves. The *uncertainty cost* represents the amount of reward lost due to robots that do not know about where worksites are located, while the *displacement cost* and the *misinformation cost* express how efficiently a swarm can turn information about worksites into reward. Displacement cost is incurred by robots that are informed about where to forage from but are unable to act on this information immediately (for example, they may be recruited to a far away worksite, or they may be recruited while they are part-way through completing another task). Misinformation cost is incurred by robots with outdated information, that are attempting to reach a worksite that has already been depleted. By measuring these costs, it is possible to identify robot behaviours that are responsible for an observed swarm performance (for instance, recruitment far away from worksites). Consequently, it is possible to identify the effect of design patterns on swarm-level behaviour, as well as how their suitability and effectiveness are affected by the parameter values of robot behaviour and by other design patterns.

## 3. Methods

### 3.1. Design Pattern Specification

Inspired by object-oriented design pattern principles (Gamma et al., 1994, p.11–42), it is proposed here that a swarm robotic design pattern should:

- Describe a particular **stand-alone module of a robot control algorithm** in terms of *robot behaviours*, relevant internal and external *data structures* and *relationships* between them. Such a module should satisfy a particular functional requirement and its description should be independent of other modules that deal with other requirements.
- Provide a description of **suitable environments and swarm tasks**, in which the pattern is understood to be an appropriate design choice.
- Be possible to **combine** with other design patterns.
- Be **implementation-generic**, i.e., only describe high-level behaviour, rather than an implementation^[2]^.

The information exchange patterns presented here are split into two categories that identify the pattern roles (see also, e.g., Gamma et al., 1994; Aridor and Lange, 1998):

- **Information Transmitter patterns:** Specify what entity transmits or stores information, as well as what information is used by behaviours and under what conditions
- **Information Aggregation patterns:** Specify where information exchange takes place and what behaviours are responsible for the exchange

Each design pattern includes the following properties (as in, e.g., Gamma et al., 1994; De Wolf and Holvoet, 2007; Gardelli et al., 2007; Fernandez-Marquez et al., 2013):

- Design pattern **name** and **category**
- The **problem** that the pattern is solving
- The **applicability** of the pattern, given the conditions of the swarm task and of the environment, as well as the available hardware
- The **solution**, including a representation of relevant robot behaviours and data structures in BDRML, as well as guidance on the pattern implementation
- A description of **feedback loops** that are created or altered as a result of using the pattern
- A list of **parameters** associated with the specified robot behaviours and their detailed description
- A list of **forces** that affect the pattern’s effectiveness
- A list of **consequences** that the design pattern has on macro-level swarm characteristics, especially those that cannot be controlled through the pattern’s parameters
- A list of **known uses** in the swarm robotics literature
- A list of **related patterns**

The description of pattern feedback loops, parameters, forces and consequences utilises terminology of the ICR framework and considers a variety of experiments reported in the literature.

Not all pattern descriptions include all of the properties listed above. For example, when a pattern has no parameters (e.g., the Individualist pattern in Section 4.1), it is likely to also not have any forces associated with it. In other cases, a solution that a pattern offers may look rather generic (for example, the Information Exchange Anywhere pattern from Section 4.4). This can happen when a pattern represents an alternative to other, more restrictive, patterns from the same category. An example of how such pattern is used is provided in Section 5.2.

Note that unlike in some design pattern work (e.g., Gamma et al., 1994; Gardelli et al., 2007; Fernandez-Marquez et al., 2013), aliases are not provided with the patterns presented here. The term “alias” implies direct correspondence, which, to the best of our knowledge, is not possible to make with other design patterns found in the literature. Instead, design patterns similar to those presented here are mentioned in the list of related patterns. Also, example code for the patterns is not provided in this paper due to content length restrictions. However, detailed high-level guidelines on implementation are provided in the form of BDRML diagrams. Furthermore, example pseudocode of four robot control algorithms that can be created by using the Design Pattern Catalogue is shown in Section 5.

### 3.2. Design Pattern Application Rules

The usage of different design patterns in a robot control algorithm can be facilitated by identifying Application Rules for modifying and combining the BDRML pattern representations. *Compulsory Rules* (C), represent a minimal set of steps for combining patterns and should always be applied. Other rules are optional, and include those for *extending* (EXT) and *redefining* (RDF) information processing routines of patterns, and those for *concretising* (CNC) the patterns.

The Compulsory Application Rules include:

- *C1.* Relabel any elements of the selected design patterns that are ambiguous with respect to one another or to the control algorithm that is being created. For instance, identify any equivalences between design pattern elements that appear in more than one of the original design patterns (e.g., two design patterns may each involve reading the same external environmental data but allocate it a different identifier). Analogously, identify any distinctions between labels that appear in more than one of the original design patterns (e.g., two design patterns may each employ the identifier “Worksite”, without this label necessarily referring to the same entity). Finally, rename primitives in order to better facilitate understanding of the control algorithm, especially if the original design patterns use general labels, such as “Worksite data”.
- *C2.* Copy all sets of behaviours, $B_{i}$, from all patterns into a new behaviour set, $B^{\prime }$, i.e., $B^{\prime }=\{B_{1}\cup B_{2}\cup \ldots \cup B_{n}\}$.
- *C3. *Copy common data structures from design pattern data structure sets $D_{i}$ and $D_{e}$ into new sets, $D_{i}$ and $D_{e}$, i.e., $D_{i}=\left\{D_{i1}\cap D_{i2}\cap \ldots \cap D_{in}\right\},D_{e}=\left\{D_{e1}\cap D_{e2}\cap \ldots \cap D_{en}\right\}.$ Choose appropriate data types if the design pattern data structures define multiple data type options.
- *C4. *Copy all relations between the primitives that belong to sets $B^{\prime }$, $D_{i}$ and $D_{e}$, including their conditions. Unless it is otherwise specified by a relation condition, assume the *or* operator when combining conditions. All conditions with the *or* operator should be considered optional^[3]^. Additionally, when the “always” condition is combined with others, the “always” condition should be deleted^[4]^.

The rules for *extending information processing routines* of a pattern by another include:

- *EXT1.* Add additional data structures into $D_{i}$*'* and $D_{e}$*'* from an included Information Transmitter pattern that have a *read* relation with a behaviour, or a *copy* relation with a data structure already present in $D_{i}$*'* or $D_{e}$*'*. This allows one pattern to extend the list of information processing routines of another, while ensuring that Information Transmitter patterns play a decisive role in what data structures are used in the robot program.
- *EXT2*. Apply Rule C4.

The rules for *redefining information processing routines* of a pattern by another are:

- *RDF1*. Delete all relations that belong to shorter *relation paths* between behaviours and data structures (but not between behaviours) that are of the same type (for example, *send* relations). A relation path specifies a set of relations that lead from a primitive $V_{1}$ to a primitive $V_{2}$, including those relations that pass through other primitives and create an indirect relation between $V_{1}$ and $V_{2}$.
- *RDF2.* If applicable, use conditions from deleted relation paths on the relation paths that are left over. For example, if relation between $V_{1}$ and $V_{2}$ with a condition c was deleted, and a new relation between $V_{3}$ and $V_{2}$ exists instead, it may be appropriate to add condition c to this relation.

Finally, patterns can be *concretised* in order to better describe the dynamics of an implemented robot control algorithm by using the following rule:

- *CNC1*. Add additional specifications to *write*, *send* and *update* relations in order to identify how they change the values of their corresponding data structures (see Figure 2).

**Figure 5:**
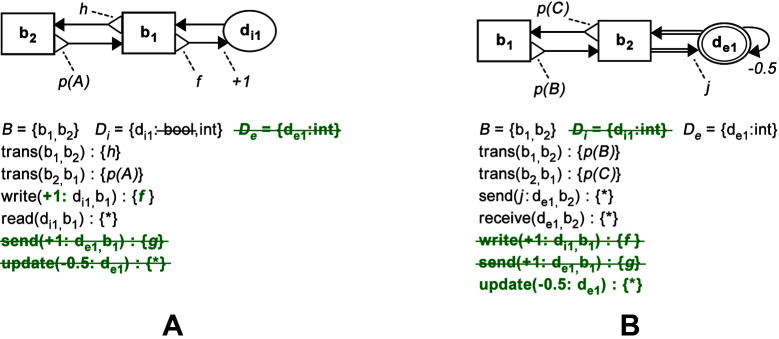
BDRML examples of combining design patterns. **(A)** Combination of design patterns from Figure 4A,C. **(B)** Combination of design patterns from Figure 4B,C. Primitives and relations of the first pattern are shown in black text. Additional primitives and relations, drawn from the second pattern in Figure 4C, are shown in bold green text. Primitives and relations that belong to one of the patterns but are not included in the control algorithm are shown as strikethrough text, but they are not shown graphically.

Consider an example in Figure 5A, where the patterns from Figure 4A,C are combined. First, a set of behaviours that belong to both patterns is found (Rules C1 and C2). This set includes the behaviours $b_{1}$ and $b_{2}$. Next, the data structure $d_{i1}$, that belongs to both patterns, is included and its data type is chosen to be integer (Rules C1 and C3). Finally, all relations that belong to $b_{1}$, $b_{2}$ and $d_{i1}$ are copied and combined (Rule C4). Note that the pattern from Figure 4C cannot extend information processing routines defined in Figure 4A, since the additional data structure from Figure 4C, $d_{e1}$, does not have a *read* or *copy* relation with another primitive (Rule EXT1). The point of including a data structure without *read* operations in a pattern, such as that in Figure 4C, is to specify special conditions for their *write*, *send* or *update* relations, that are applicable in cases when the pattern is combined with another pattern, in which the data structure is read or copied.

An example of information processing redefinition is shown in Figure 5B, which results from combining patterns from Figure 4B and C. Primitives $b_{1}$, $b_{2}$ and $d_{e1}$, as well as their relations are copied over (Rules C1–C4). In order to use the design pattern from Figure 4C to redefine information processing described in Figure 4B, the *write* relation between $b_{1}$ and $d_{e1}$ is deleted, as it represents a shorter relation path (Rule RDF1).

Apart from a BDRML representation of the robot behaviour, other characteristics of design patterns should be considered together when design patterns are combined. The list of suitable applications becomes more specific when multiple patterns form a control algorithm. Or, from another point of view, a more detailed specification of the swarm’s environment and task allows a programmer to choose between design patterns with a higher confidence. The list of control algorithm parameters also grows when multiple patterns are combined. Therefore, in order to minimise the number of design decisions that need to be made, design patterns with a smaller number of parameters should be preferred where possible. Unless an exhaustive list of situations is considered during a control algorithm optimisation phase, or unless a suitable on-line parameter learning algorithm is implemented, each new parameter can lead to undesirable dynamics.

## 4. The Design Pattern Catalogue

In this section, six information exchange design patterns for robot swarm foraging are presented. The particular patterns were selected due to the considerably large number of detailed simulation experiments that we have previously performed using robot controllers with behaviours described by these patterns (Pitonakova et al., 2014, 2016a, b, Pitonakova et al., 2018). Also, as we will show below, each pattern can be found in control algorithms that were used in a relatively large number of other swarm robotics research papers.

The term “worksite” is used to refer to a place where reward is located, such as a place where items need to be collected from [e.g., during raw material collection, package delivery, etc. (as in, e.g., Winfield, 2009a; Wawerla and Vaughan, 2010)], or to a place where work needs to be performed [e.g., during shop floor machine maintenance (e.g., Sarker and Dahl, 2011) or similar tasks (e.g., Jevtic et al., 2012)].

### 4.1. Individualist

#### 4.1.1. Category

Information Transmitter pattern.

#### 4.1.2. Problem

Robots need to find and exploit worksites as quickly as possible.

#### 4.1.3. Applicability

Information about worksites is easily obtainable, for example when worksite density is high (Winfield, 2009a; Pitonakova et al., 2014, 2016a, Pitonakova et al., 2018). Also recommended when continuous exploration of the environment is important, e.g., when new worksites appear over time (Pitonakova et al., 2018).

#### 4.1.4. Solution (see also Figure 6)

A robot scouts for worksites in the environment. A successful scout stores information about its worksite, such as its location, in an internal data structure (“Worksite data int.”), and begins work. The data structure may be updated and utilised periodically while the robot works. For example, if the robot uses odometry to localise itself relative to the worksite, the relative vector to the worksite should be updated periodically. The robot ignores any information and actions of other members of the swarm.

**Figure 6:**
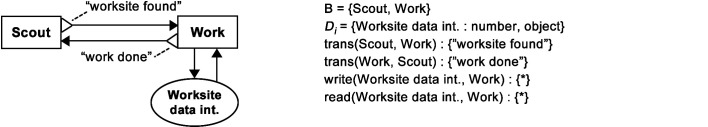
BDRML representation of the Individualist design pattern.

#### 4.1.5. Feedback Loops: -

#### 4.1.6. Parameters: -

#### 4.1.7. Forces: -

#### 4.1.8. Consequences

- Leads to a low information gain rate, which is why information about worksites needs to be relatively easy to find (Pitonakova et al., 2018)
- Minimises displacement and misinformation costs (Pitonakova et al., 2018)
- The spread of robots across worksites only depends on their scouting movement pattern. For example, an even spread across the environment may be achieved when robots utilise random walk (Sugawara and Watanabe, 2002; Kernbach et al., 2012; Pitonakova et al., 2018)
- Prevents the spread of erroneous information among robots (Pitonakova et al., 2014)

#### 4.1.9. Known Uses

Often used when simple foraging algorithms are needed as a basis for robot behaviour, while other swarm behaviours, such as self-regulation or task-allocation, are explored (Krieger and Billeter, 2000; Labella et al., 2006; Lerman et al., 2006; Campo and Dorigo, 2007; Kernbach et al., 2012). Also used in studies that compare swarms that do and do not utilise robot-robot recruitment (Balch and Arkin, 1994; Rybski et al., 2007; Gutiérrez et al., 2010; Lee et al., 2013; Fujisawa et al., 2014; Amato et al., 2015) and in scenarios where robots can infer information about others and about the environment through sensing (e.g., by using a camera), rather than through communication (Jones and Mataric, 2003).

#### 4.1.10. Related Patterns

Serves an alternative to the Broadcaster and Information Storage patterns, that, in general, is easier to implement and provides collective performance that is less difficult to understand due to the lack of parameters.

### 4.2. Broadcaster

#### 4.2.1. Category

Information Transmitter pattern.

#### 4.2.2. Problem

Robots need to find and exploit worksites as quickly as possible, but the task characteristics (e.g., robot or worksite density) make it difficult for robots to discover worksites.

#### 4.2.3. Applicability

Robots are capable of directly communicating with each other (as in, e.g., Wawerla and Vaughan, 2010; Ducatelle et al., 2014; Pitonakova et al., 2018).

#### 4.2.4. Solution (see also Figure 7)

A robot scouts for worksites in the environment. A successful scout stores information about its worksite, such as its location, in an internal data structure (“Worksite data int.”), and begins work. The data structure may be updated and used periodically while the robot works. A robot that is engaged in the “Work” behaviour may send information about its worksite to another robot, provided that a boolean *recruitment* function, r, returns *true*. When a robot receives worksite data in this way, it stores it in its own internal data structure and transitions from the “Scout” to the “Work” behaviour, provided that a boolean *adoption* function, a, returns *true*.

**Figure 7:**
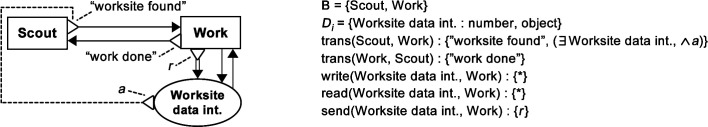
BDRML representation of the Broadcaster design pattern.

#### 4.2.5. Feedback Loops

Sharing of worksite information represents a positive feedback loop that can be regulated via the pattern’s parameters.

#### 4.2.6. Parameters

- Recruitment function, r: A boolean function that determines whether the robot decides to recruit another robot. For example, a robot might decide to recruit with a certain probability every time it encounters another robot
- Adoption function, a: A boolean function that determines whether a scout transitions to the “Work” behaviour after receiving worksite information. For example, a robot might prefer worksites from a certain area only.
- Robot communication range: A range at which robots can communicate with one another

#### 4.2.7. Forces

- A sufficient communication range must be available in order for recruitment to take place, depending on the worksite and robot density (Sugawara and Watanabe, 2002).
- A larger communication range causes a higher information gain rate, but can also increase displacement and misinformation costs incurred by recruited robots, consequently decreasing the swarm performance (Sugawara and Watanabe, 2002; Valdastri et al., 2006; Rybski et al., 2007; Pitonakova et al., 2016a).

#### 4.2.8. Consequences

- Information about worksites is more easily accessible to uninformed robots (Sugawara and Watanabe, 2002; Rybski et al., 2007; Sarker and Dahl, 2011)
- Information is carried and transmitted by robots, meaning that the information gain rate depends on the probability of robots meeting each other, i.e., on their movement algorithm and on the structure of the environment (Pitonakova et al., 2018)
- Causes the robots to incur displacement cost, associated with traveling to worksites after being recruited (Pitonakova et al., 2018)
- Increases the probability of incurring misinformation cost, as a result of outdated information potentially being spread across the swarm (Gardelli et al., 2007; Fernandez-Marquez et al., 2013; Pitonakova et al., 2018)
- Can lead to the spread of erroneous information among robots, e.g., when a recruiter’s worksite information is incorrect due to sensory-motor noise (Pitonakova et al., 2014)

#### 4.2.9. Known Uses

Often used to implement local communication of robot state (Balch and Arkin, 1994; Parker, 1995; Dahl, 2002; Sugawara and Watanabe, 2002; Rybski et al., 2007), worksite location (Balch and Arkin, 1994; Parker, 1995; Valdastri et al., 2006; Wawerla and Vaughan, 2010; Sarker and Dahl, 2011; Amato et al., 2015; Pitonakova et al., 2018) and worksite urgency (Sarker and Dahl, 2011). It has been used in tasks like general (Balch and Arkin, 1994; Pitonakova et al., 2018) and central-place (Parker, 1995; Valdastri et al., 2006; Rybski et al., 2007; Pitonakova et al., 2018) foraging, cooperative transportation (Sugawara and Watanabe, 2002; Amato et al., 2015), package delivery (Wawerla and Vaughan, 2010) and task allocation (Sarker and Dahl, 2011).

#### 4.2.10. Related Patterns

The pattern can be combined with the Information Exchange Anywhere pattern to make robots exchange information at any time they meet in the foraging arena (Gutiérrez et al., 2010; Fraga et al., 2011). When combined with Information Exchange near Worksites pattern, a behaviour similar to that of sheep (Michelena et al., 2010) and fish (Lachlan et al., 1998) is obtained, where robots that are currently obtaining reward from worksites attract nearby robots, effectively increasing the worksite detection range (e.g., as in Wawerla and Vaughan, 2010; Ducatelle et al., 2014; Pitonakova et al., 2018). Finally, bee-inspired recruitment (Seeley et al., 1991), that involves communication in the base, can be obtained by combining the Broadcaster and the Information Exchange Centre pattern (e.g., as in Parker, 1995; Krieger and Billeter, 2000; Pitonakova et al., 2018).

Other related patterns include “Diffusion” (Gardelli et al., 2007) and “Spreading” (Fernandez-Marquez et al., 2013).

### 4.3. Information Storage

#### 4.3.1. Category

Information Transmitter pattern.

#### 4.3.2. Problem

Robots need to find and exploit worksites as quickly as possible, but the task characteristics (e.g., robot or worksite density) make it difficult for robots to discover worksites.

#### 4.3.3. Applicability

Robots are capable of depositing information into their environments and retrieving it, for example to drop, update and read RFID tags (Drogoul and Ferber, 1993; Hrolenok et al., 2010), deposit and sense chemicals (Mayet et al., 2010; Fujisawa et al., 2014), or store and read “virtual pheromone” maintained by stationary robots (Hoff et al., 2010; Ducatelle et al., 2011) or by an external server (Sugawara et al., 2004; Kazama et al., 2005).

#### 4.3.4. Solution (see also Figure 8)

A robot scouts for worksites in the environment. Additionally, it can adopt information about a worksite if it finds a *data storage device* containing information (“Worksite data ext.”), and if a boolean *adoption function*, a, returns *true*. Once a robot discovers information about a worksite either as a result of scouting or when finding a data storage device, it begins work. The robot’s internal data structure is used and updated with information stored in the environment while the robot works, based on a. An informed robot deposits information about its worksite into data storage device(s) when appropriate.

**Figure 8:**
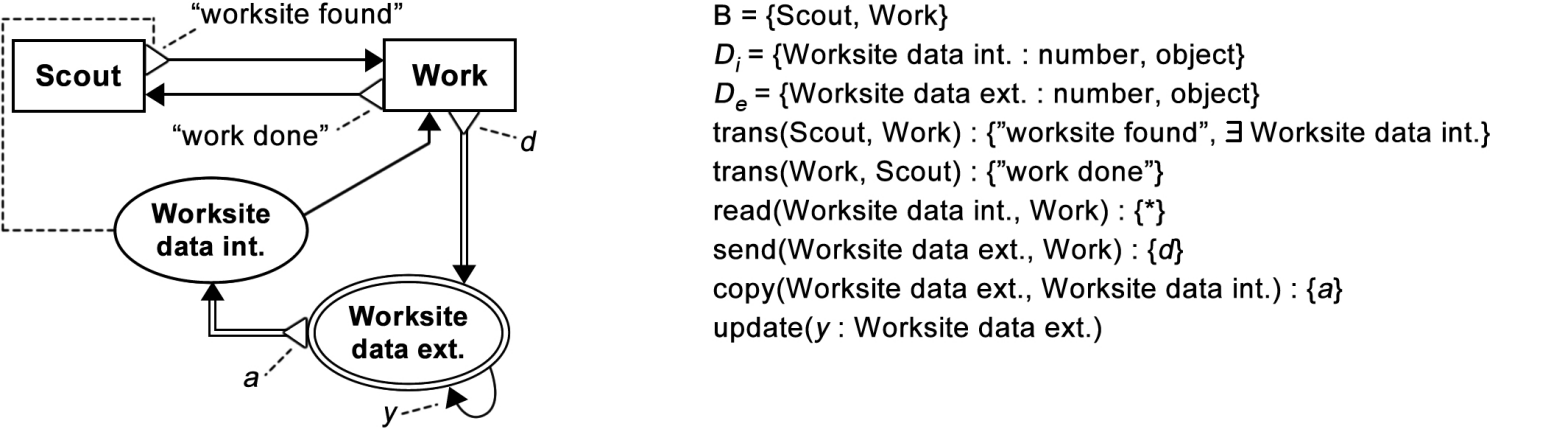
BDRML representation of the Information Storage design pattern.

#### 4.3.5. Feedback Loops

Depositing information in the environment acts as a positive feedback loop that can be regulated via the pattern’s parameters. Additionally, a negative feedback loop can be created by applying the *update* relation to “Worksite data ext.”.

#### 4.3.6. Parameters

- Deposit function, d: A boolean function that determines whether a robot deposits information into an external data structure. For example, a robot might deposit a chemical trail any time it is traveling between a worksite and the base (Mayet et al., 2010; Fujisawa et al., 2014). In other cases, a robot might decide to drop an RFID tag into the environment based on a perceived density of RFID tags nearby (Hrolenok et al., 2010).
- Adoption function, a: A boolean function that determines whether a robot copies information that it finds in a data storage device into its own internal data structure. For example, a robot might only adopt external data when it does not have any worksite information, or when the external data is more up-to-date (Hecker and Moses, 2015).
- Decay function, y: A function according to which information in the data storage device(s) is updated. For example, if “Worksite data ext.” stores a vector towards a worksite location, y can determine how up-to-date the information is (Hecker and Moses, 2015). On the other hand, if ‘Worksite data ext.” is a real number, as is often the case in pheromone-inspired algorithms, y can define how the real value “evaporates” over time (Hrolenok et al., 2010; Fujisawa et al., 2014).
- Detection range: A range at which a robot can find storage devices.

#### 4.3.7. Forces

- The decay function affects how long the information about worksites remains available in each storage device, i.e., the *lifespan* of the stored information. The function therefore must consider dynamics of the environment. An information life span that is too long causes robots to get recruited to depleted worksites and incur high misinformation cost, while a very short information life span prevents robots from utilising the stored information (Drogoul and Ferber, 1993; Garnier et al., 2007).

#### 4.3.8. Consequences

- Information about worksites is more easily accessible to uninformed robots. Information gain rate depends on the probability of robots to detect the information storage devices, but not on the probability of robots to meet each other (Pitonakova et al., 2018).
- Causes robots to incur displacement and misinformation costs, as a result of recruitment to worksites. The extent of these costs increases with an increasing robot density as a result of congestion (Drogoul and Ferber, 1993; Parker, 1995; Hoff et al., 2010).
- Can lead to the spread of erroneous information through the swarm, e.g., when a depositing robot’s worksite information is incorrect due to sensory-motor noise (Hecker et al., 2012).

#### 4.3.9. Known Uses

Often used for studying ant-inspired central-place foraging behaviour (Beekman et al., 2001), where “pheromone” trails are formed between the robot base and worksites, helping the robots to navigate an unknown environment (Drogoul and Ferber, 1993; Sugawara et al., 2004; Kazama et al., 2005; Hoff et al., 2010; Hrolenok et al., 2010; Mayet et al., 2010; Ducatelle et al., 2011; Fujisawa et al., 2014). Has also been used for robot recruitment in a cooperative transportation task (Amato et al., 2015).

#### 4.3.10. Related Patterns

Depending on the type of storage device used, the pattern can either be combined with the Information Exchange Anywhere pattern, in order to form pheromone trails made of RFID tags (Drogoul and Ferber, 1993; Hrolenok et al., 2010), chemicals (Mayet et al., 2010; Fujisawa et al., 2014) or by other means (Sugawara et al., 2004; Kazama et al., 2005; Hoff et al., 2010; Ducatelle et al., 2011), or with the Information Exchange Centre pattern, in order to store information about worksites in the robot base (Alers et al., 2011; Amato et al., 2015; Hecker and Moses, 2015).

Other related patterns include “Evaporation” (Gardelli et al., 2007; Fernandez-Marquez et al., 2013), “Diffusion” (Gardelli et al., 2007), “Gradient”, “Digital pheromone” and “Ant foraging” (Fernandez-Marquez et al., 2013)

### 4.4. Information Exchange Anywhere

#### 4.4.1. Category

Information Aggregation pattern.

#### 4.4.2. Problem

Robots need to exchange information frequently.

#### 4.4.3. Applicability

Robots are able to either directly communicate with each other (e.g., as in Wawerla and Vaughan, 2010; Ducatelle et al., 2014; Pitonakova et al., 2018) or to store information in the environment and retrieve it (Hrolenok et al., 2010; Fujisawa et al., 2014, e.g., as in).

#### 4.4.4. Solution (see also Figure 9)

Information, stored in internal or external data structures, is exchanged anywhere in the environment.

**Figure 9:**
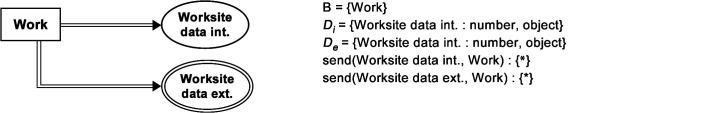
BDRML representation of the Information Exchange Anywhere design pattern.

#### 4.4.5. Feedback Loops

Positive feedback loops that already exist in the swarm behaviour are enforced.

#### 4.4.6. Parameters: -

#### 4.4.7. Forces: -

#### 4.4.8. Consequences

- Frequent exchange of information between robots can lead to a strong preference for a single worksite (Gutiérrez et al., 2010; Fraga et al., 2011). Therefore, a mechanism for regulation of recruitment may need to be implemented in order to prevent congestion and poor swarm performance.

#### 4.4.9. Known Uses

To study the problem of decentralised worksite localisation (Hoff et al., 2010; Gutiérrez et al., 2010; Fraga et al., 2011; Fujisawa et al., 2014) and decentralised task allocation algorithms (Sarker and Dahl, 2011).

#### 4.4.10. Related Patterns

Serves an alternative to the Information Exchange near Worksites and the Information Exchange Centre patterns, where the robot behaviour may be easier to understand due to the lack of parameters, but where the swarm performance might deteriorate as a result of frequent information sharing.

Often used in combination with the Information Storage pattern to create chemical or other trails between the base and worksites in central-place foraging (Drogoul and Ferber, 1993; Kazama et al., 2005; Hoff et al., 2010; Hrolenok et al., 2010; Mayet et al., 2010; Ducatelle et al., 2011; Fujisawa et al., 2014). Can also be used in combination with the Broadcaster pattern in order to allow robots to exchange information locally (Gutiérrez et al., 2010; Fraga et al., 2011; Sarker and Dahl, 2011).

### 4.5. Information Exchange Near Worksites

#### 4.5.1. Category

Information Aggregation pattern.

#### 4.5.2. Problem

The information exchange of robots needs to be regulated.

#### 4.5.3. Applicability

Uninformed robots are likely to encounter data transmitters, i.e., other robots or non-robot data storage devices, near worksites, for example, when robots remain near worksites for a sufficient amount of time, when worksite and/or robot density are high or when robots have a large communication range (Pitonakova et al., 2018).

#### 4.5.4. Solution (see also Figure 10)

Robots only exchange information while they are near worksites. Note that in the BDRML syntax, the conditions of the two relations, that connect the “Work” behaviour with the “Worksite data int.” and “Worksite data ext.” data structures, have an “and” operator. This ensures that the conditions always have to be met when this design pattern is combined with other patterns, allowing this pattern to regulate positive feedback loops of others.

**Figure 10:**
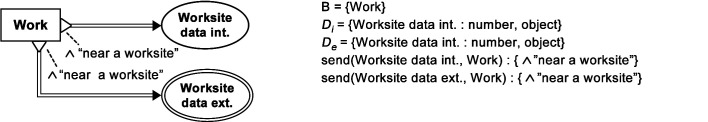
BDRML representation of the Information Exchange near Worksites design pattern.

#### 4.5.5. Feedback Loops

Positive feedback loops already present in the swarm behaviour are regulated by only allowing information exchange near worksites.

Positive feedback loops already present in the swarm behaviour are regulated by only allowing information exchange near worksites.

#### 4.5.6. Parameters

- Proximity threshold: Maximum distance at which a robot is considered to be “near a worksite”.

#### 4.5.7. Dependencies

- The proximity threshold value represents a trade-off between how much displacement and misinformation cost the robots will incur and how much recruitment can take place. If the threshold is large, robots can recruit while being further away from worksites, and thus cover a larger recruitment area, but new recruits incur larger costs.

#### 4.5.8. Consequences

- After an initial worksite discovery by a robot, the range at which other robots can find the worksite is enlarged, increasing the swarm’s scouting success (Sugawara and Watanabe, 2002; Sarker and Dahl, 2011; Pitonakova et al., 2018)
- The information gain rate depends on the structure of the environment, especially on worksite density, and on the range at which data transmitters can be detected and communicated with (Pitonakova et al., 2018)

#### 4.5.9. Known Uses

Has been used to extend the range at which robots sense worksites during foraging (Sugawara and Watanabe, 2002; Pitonakova et al., 2018), package delivery (Wawerla and Vaughan, 2010) and general event-servicing (Ducatelle et al., 2014).

#### 4.5.10. Related Patterns

An alternative to the Information Exchange Anywhere pattern, providing information flow regulation by localising information sharing to areas around worksites. Usually combined with the Broadcaster pattern to achieve foraging behaviour similar to that of as sheep (Michelena et al., 2010) and fish (Lachlan et al., 1998), where a foraging robot attracts more foragers that are nearby (Sugawara and Watanabe, 2002; Wawerla and Vaughan, 2010; Ducatelle et al., 2014; Pitonakova et al., 2018).

### 4.6. Information Exchange Centre

#### 4.6.1. Category

Information Aggregation pattern.

#### 4.6.2. Problem

The information exchange of robots needs to be regulated, or, robots have a low probability of meeting each other during foraging due to low density of worksites and/or robots.

#### 4.6.3. Applicability

Robots are able to navigate sufficiently long distances without significantly distorting their private information about worksites, e.g., as a result of cumulative effect of sensory-motor noise, which could result in incorrect information being passed to others (Pitonakova et al., 2014). Especially applicable during central-place foraging, provided that the Information Exchange Centre is identical to the place where robots need to travel to periodically in order to drop off resource (Dornhaus et al., 2006; Lemmens et al., 2008; Bailis et al., 2010; Pitonakova et al., 2014, 2016a, Pitonakova et al., 2018).

#### 4.6.4. Solution (see also Figure 11)

Robots meet at the Information Exchange Centre (IEC) in order to exchange information. There are two types of robots found at the IEC: informed robots, that provide information and uninformed robots that search for information. An informed robot pauses its work and returns to the IEC when its boolean *recruitment initiation function, i, *returns *true*, in order to begin providing information at the IEC. The robot leaves the IEC based on a *recruitment expiry function*, e, and resumes work.

**Figure 11:**
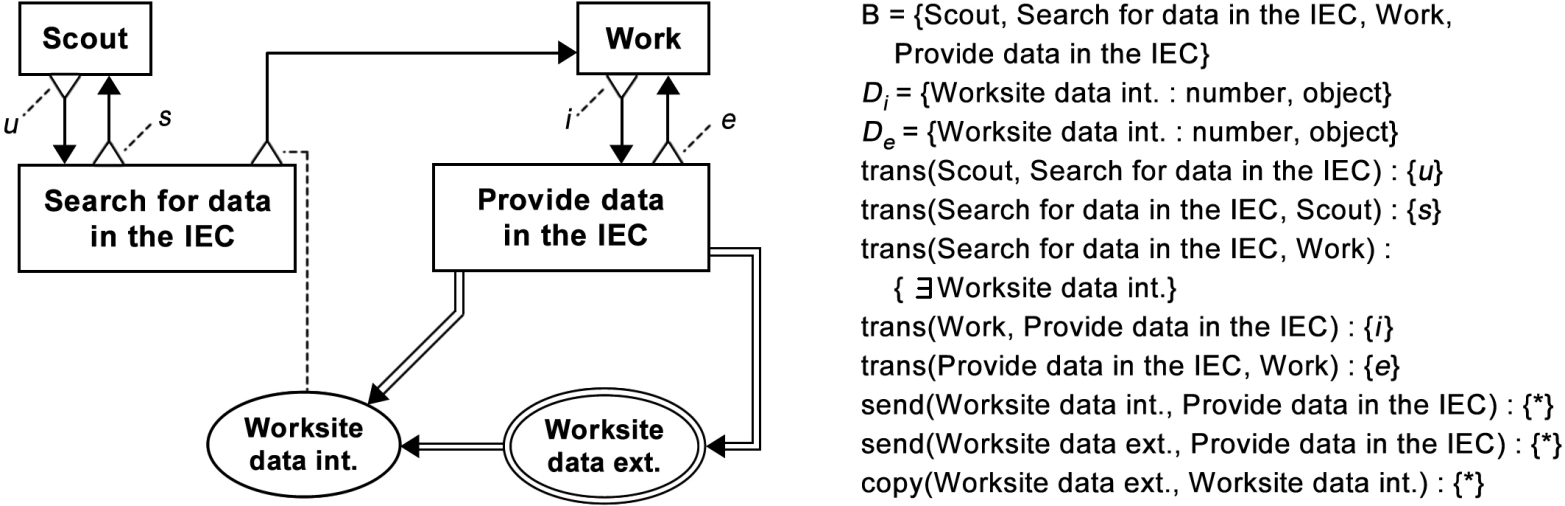
BDRML representation of the Information Exchange Centre design pattern.

An uninformed robot located outside of the IEC, i.e., a scout, returns to the IEC based on a *scouting expiry function*, u, in order to check whether new information is available. If the robot finds information about where work is located, either as a result of robot-robot recruitment, or after adopting data available in a non-robot entity, it transitions to the “Work” behaviour and leaves the IEC. If no information is available in the IEC, the uninformed robot resumes scouting when a *scouting initiation function*, s, returns *true*.

Note that the relations between the data structures and other primitives have an always condition. This signifies the fact that IEC is an exchange pattern and its role is therefore to identify where information exchange takes place, but not the conditions under which information is utilised by behaviours.

#### 4.6.5. Feedback Loops

Depending on the context within which it is used and on the selected parameter values, this pattern can either enforce positive feedback loops that already exist in the swarm behaviour by designating an area where robots are likely to find information, or provide regulation of information transfer by forcing robots to travel to a designated location in order to exchange information.

#### 4.6.6. Parameters

- Transmission initiation function, i: a boolean function that determines whether an informed robot returns to the IEC. For example, a robot might need to drop off resource during central-place foraging (Krieger and Billeter, 2000; Hecker and Moses, 2015).
- Transmission expiry function, e: a boolean function that determines whether an informed robot leaves the IEC. For example, if the IEC pattern is combined with the Broadcaster pattern, expiry of a recruitment time can trigger a robot to resume work (Pitonakova et al., 2016a; Valentini et al., 2016).
- Scouting expiry function, u: a boolean function that determines whether a scout returns to the IEC. For example, when the robot spends a certain amount of time scouting unsuccessfully (Pitonakova et al., 2016a, Pitonakova et al., 2018).
- Scouting initiation function, s: a boolean function that determines whether an uninformed robot in the IEC becomes a scout. For example, the robot might do so with a certain probability each second (Pitonakova et al., 2016a, Pitonakova et al., 2018), or when demand for resources reaches a threshold (Krieger and Billeter, 2000).

#### 4.6.7. Forces

- The scouting efficiency of the swarm decreases due to the fact that scouts return to the IEC. The scouting expiry function, u, thus must fit the nature of the environment. For example, enough time must be given to scouts to explore a large or a dynamic working area, while at the same time ensuring that robots do not spend too much time outside of the base, where information may be readily available (Pitonakova et al., 2018).
- The swarm size and its relation to the area of the IEC play an important role, since a large number of robots situated in the IEC at the same time can cause congestion and decrease the swarm performance (Lee et al., 2013; Pitonakova et al., 2016b).

#### 4.6.8. Consequences

- Information gain rate is less dependent on the structure of the environment, on the communication range of robots and on the robot movement algorithm. The variance in information gain rate is small across different environments (Pitonakova et al., 2018).
- Promotes spatio-temporal coordination between robots. This is advantageous when a single worksite exists in the environment. On the other hand, the swarm performance is poor when the swarm needs to concentrate on multiple worksites simultaneously (Krieger and Billeter, 2000; Pitonakova et al., 2018).
- The amount of the incurred misplacement and misinformation costs depends on the structure of the environment, especially on the worksite distance from the IEC. A larger worksite distance generally leads to higher costs being incurred (Pitonakova et al., 2018).

#### 4.6.9. Known Uses

Most prominently used to study bee-inspired (Seeley et al., 1991) multi-robot foraging algorithms (Krieger and Billeter, 2000; Pitonakova et al., 2014; Hecker and Moses, 2015; Reina et al., 2015; Pitonakova et al., 2016a, Pitonakova et al., 2018), where robots collect items from the environment and return them to the base, where they also recruit in a peer-to-peer fashion. It has also been used to help robots recruit each other in the base during a cooperative transportation task (Amato et al., 2015).

#### 4.6.10. Related Patterns

Provides an alternative to the Information Exchange Anywhere and Information Exchange near Worksites patterns, by making the ability of robots to share information less dependent on the effectiveness of robot communication hardware and on the task parameters.

The pattern can be either combined with the Broadcaster pattern, in order to facilitate local interactions of agents in the base (Krieger and Billeter, 2000; Pitonakova et al., 2014, 2016a, Pitonakova et al., 2018), or with the Information Storage pattern, in order to turn the base into a repository of information that robots can read from without the need to meet each other (Alers et al., 2011; Hecker et al., 2012; Amato et al., 2015).

A related pattern that involved bee-inspired collective decision-making has been described in (Reina et al., 2015).

## 5. Applications

After considering the properties of design patterns, in particular their applicability, feedback loops, forces and consequences, and matching them with information about a specific robot swarm mission and available robot hardware, a robot control algorithm can be created by utilising the Design Pattern Application Rules. Here we first show how the Individualist, Broadcaster and Information Exchange near Worksites patterns were validated on a foraging e-puck swarm and how the latter two improved the swarm performance. Further examples of how the Catalogue could be used in different real-world missions are then provided.

### 5.1. Improving Robustness to Noise in Foraging E-Puck Swarms

Five e-puck robots with the Linux extension board developed at the Bristol Robotics Laboratory (Liu and Winfield, 2011) were tasked with searching an arena for worksites and delivering virtual resources from the worksites into the base until all worksites were depleted. The arena was 2×1.5 m large and it was characterised by the number of worksites, $N_{W}\in \{1,3,12\}$ and minimum worksite distance from the base, D∈{0.7,1.4} m. The base was represented by a quarter-circle area with 0.4 m radius that was located in one of the arena’s corners. Worksites were placed randomly at a distance between D and D±0.5 m from the base edge. They were circular, with a radius of 0.1 m. The arena contained a total of 48 units of resource, where each worksite had $48/N_{W}$ units at the beginning of an experiment.

The robots navigated by obtaining their absolute coordinates from an overhead tracking system. Additionally, the robots were equipped with a virtual worksite sensor with a range of 0.25 m. When a robot was at least 0.25 m away from a worksite, it received the worksite location from an external computer, allowing the robot to calculate a direction vector towards the worksite.

**Figure 12:**
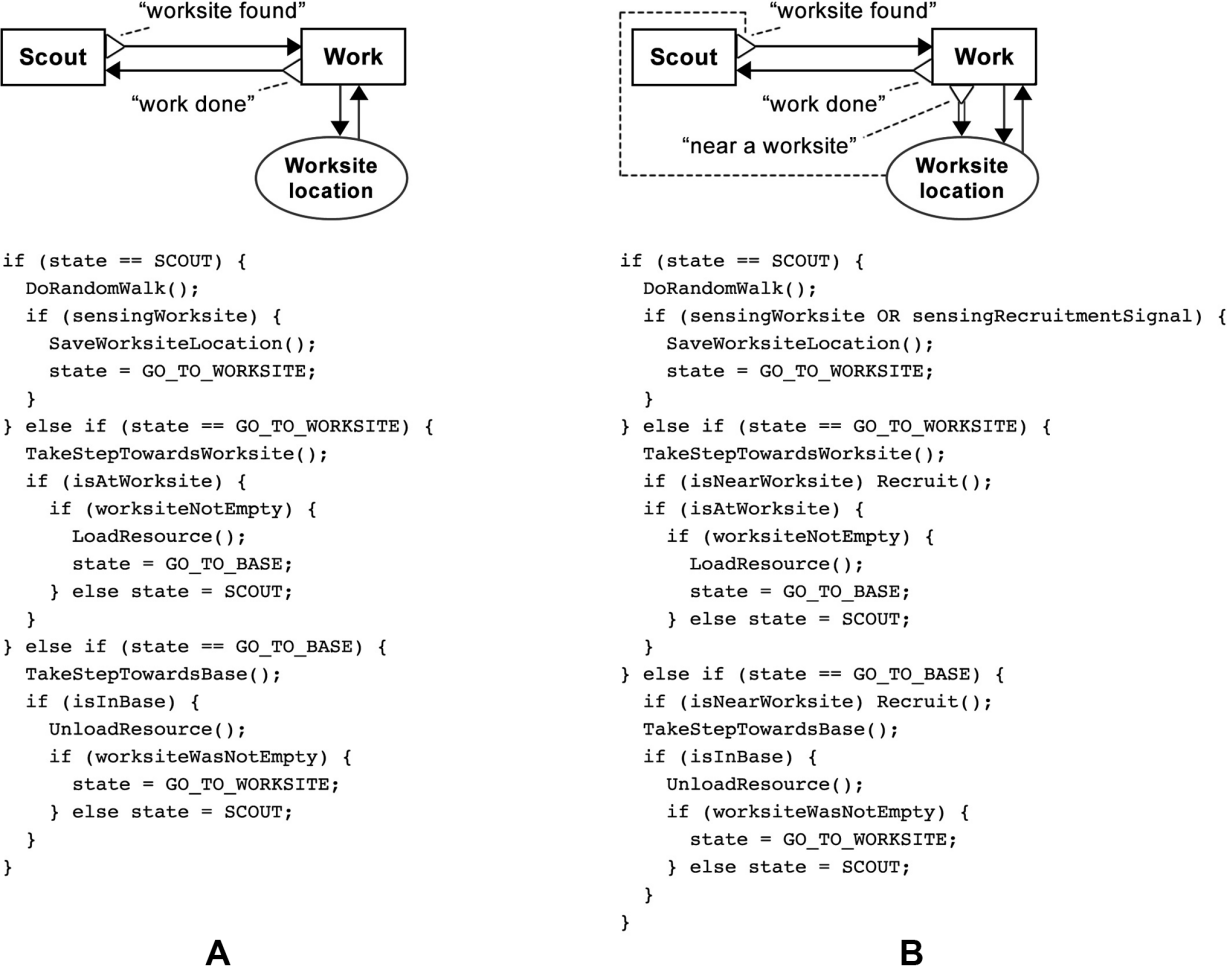
**(A)** Graphical BDRML representation of the “Solitary” robot control algorithm, resulting from the Individualist design pattern, and robot update loop pseudocode. **(B)** Graphical BDRML representation of the “Local Broadcaster” robot control algorithm, resulting from a combination of the Broadcaster and Information Exchange near Worksites patterns, and robot update loop pseudocode.

First, a “Solitary” redfinite-state machine robot controller was created based on the Individualist design pattern (Figure 12A). This involved copying the pattern’s BDRML primitives, and renaming its data structure to “Worksite location”. Note the difference between the high-level representation in BDRML and the pseudocode built for the finite state machine controller in Figure 12A. In this particular implementation, the “Work” behaviour was represented by two states, “GO_TO_WORKSITE” and “GO_TO_BASE”. If the controller was based on a neural network, for instance, a different implementation would be needed. However, in both cases, the robot would be required to adhere to the high-level BDRML specification by exhibiting the “Scout” and “Work” behaviours.

**Figure 13:**
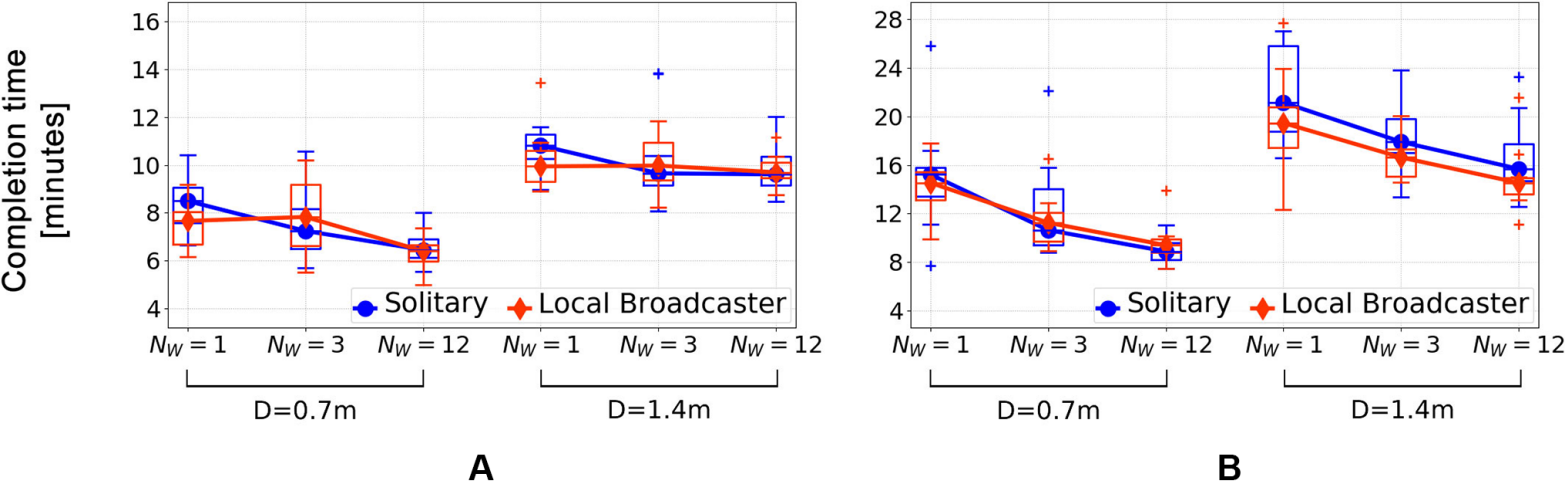
The e-puck swarm performance in environments **(A)** without and **(B)** with positional noise.

The Solitary swarm successfully collected all the resource within around 6–12 min, depending on the number of worksites and the arena size (Figure 13A). In the second set of experiments, noise was added into the positional information that the robots received from the tracking system, so that the robots could not always arrive to their worksites and had to abandon and re-discover them. In other words, the noise caused the robots to loose information about worksites, which was reflected by an increased amount of uncertainty cost paid by the swarm. Consequently, the swarm performance decreased and the robots needed more time to complete the task (Figure 13).

In order to decrease the negative effect of information loss due to noise, a “Local Broadcaster” controller was created as a result of combining the Broadcaster and the Information Exchange near Worksites (IEW) patterns (Figure 12B). The robots were equipped with a wireless communication module with a maximum range of 1.25 m. Because of the availability of this hardware, the Broadcaster pattern represented a better choice than the alternative Information Transmitter pattern, Information Storage, where additional devices or chemicals would have to be placed by the robots into the environment. The IEW pattern was selected in order to solve the robot navigation problem by increasing the range at which robots could detect worksites. The Information Exchange Centre pattern was not suitable, since it requires robots to travel relatively long distances to the base and back to worksites, potentially increasing the negative effects of positional noise. On the other hand, using the Information Exchange Anywhere pattern could prevent the swarm from designating a portion of its effort to scouting the environment, which was especially important in environments with twelve worksites.

The resulting “Local Broadcaster” controller was created by, first, copying the behaviours and common data structures that belonged to both patterns (Design Pattern Application Rules C1–C3). These sets included the “Scout” and the “Work” behaviours and the “Worksite data int.” data structure, which was renamed to “Worksite location”. The relations between all included primitives, as well as their applicable conditions, were then included (Rule C4). The recruitment function, r, and the adoption function, a, were defined in the Broadcaster pattern as *or* conditions and were therefore optional. They were not included in the control algorithm in the interest of simplicity. At the same time, “Worksite data ext.”, defined in the IEW pattern, was not included, since IEW is not an Information Transmitter pattern (Rule EXT1).

The Local Broadcaster swarm was able to maintain information about approximate worksite locations better that the Solitary swarm, which made it more robust to the positional noise (Figure 13B). When worksites were further away from the base (D=1.4 m), the completion time of the Broadcaster swarm was consistently lower than that of the Solitary swarm. Additionally, Local Broadcasters also achieved a lower completion time variance in the environment with the lowest worksite density ($N_{W}=1$, D=1.4 m).

### 5.2. Other Examples

**Figure 14:**
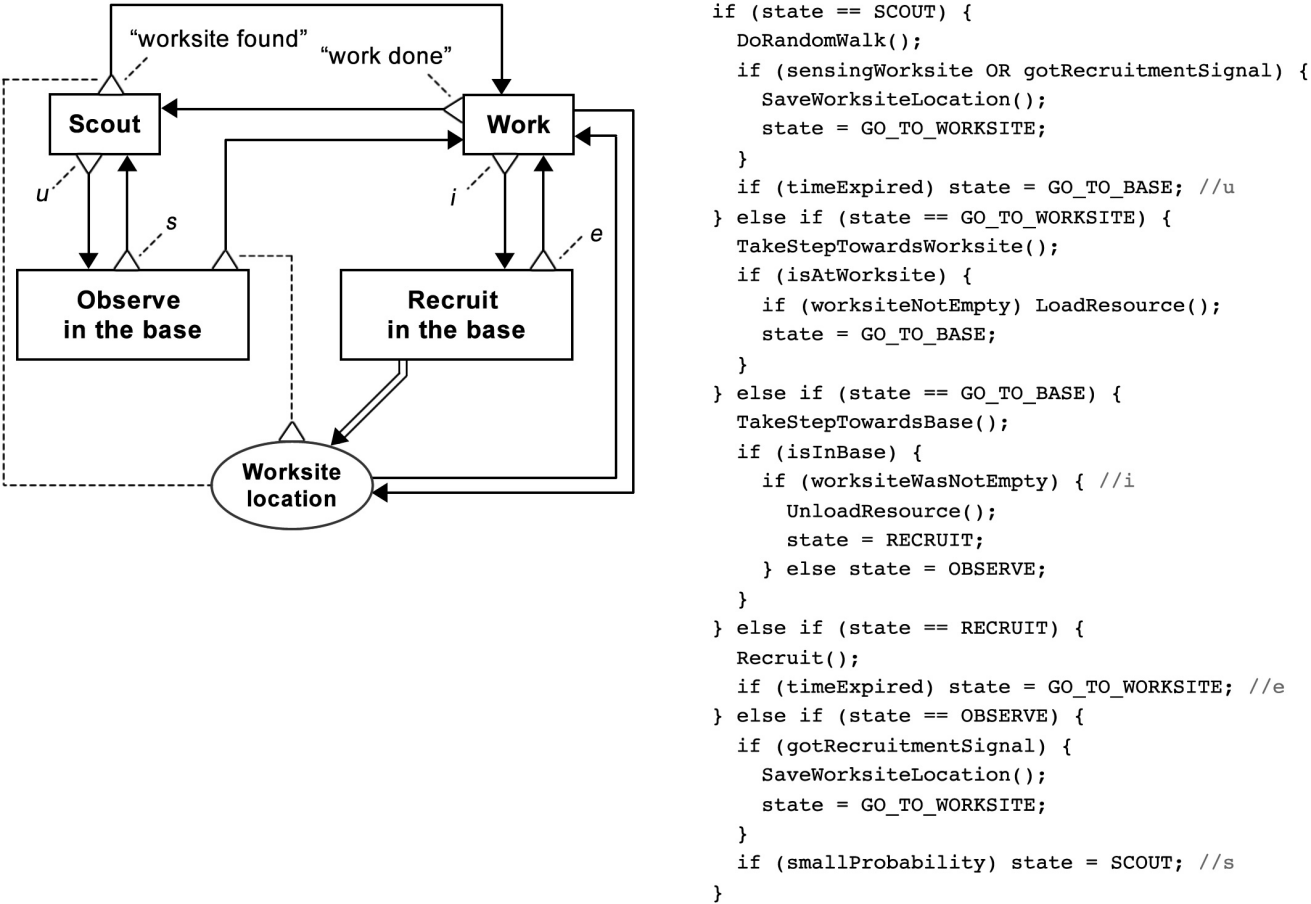
Graphical BDRML representation of the Bee-Inspired robot control algorithm, resulting from a combination of the Broadcaster and Information Exchange Centre patterns, and robot update loop pseudocode. Implementations of BDRML relation conditionshave been indicated in the pseudocode following the *//* characters.

In missions like mineral collection, robot swarms could face the challenge of discovering mineral veins of low density, while minimising the mission time would be desirable. Once a vein has been discovered, multiple robots could dedicate their effort to carrying the minerals back to a depot, in order to satisfy their output quota as quickly as possible. In this mission, creating a “Bee-Inspired” swarm (Figure 14) as a combination of the Broadcaster and Information Exchange Centre (IEC) patterns could be suitable, provided that the robots could navigate reliably over longer distances, for example by using GPS. Such an algorithm is well-known in the swarm foraging literature (Krieger and Billeter, 2000; Amato et al., 2015; Pitonakova et al., 2016a, Pitonakova et al., 2018).

In the Bee-Inspired swarm, robots that know about worksites return to a depot in order to recruit uninformed robots. Unsuccessful scouts also return to the depot and search for informed robots. The control algorithm has four behaviours and one internal data structure common to both patterns. The BDRML primitives are renamed in order to facilitate understanding of the resulting algorithm (Rule C1). Similarly as was the case with the Local Broadcaster algorithm, “Worksite data ext.” is not included in the Bee-Inspired algorithm^[5]^ because it does not satisfy the condition of Rule EXT1. Additionally, like in the Local Broadcaster algorithm, the “Work” behaviour is represented by two states in the pseudocode implementation, “GO_TO_WORKSITE” and “GO_TO_BASE”. The IEC pattern is used to redefine the communication routines of Broadcaster by applying Rules RDF1 and RDF2. The *send* relation between “Work” and “Worksite location”, defined in the Broadcaster pattern, is deleted, since a longer relation path that includes a *send* relation and passes through the “Recruit in the base” behaviour exists in the IEC pattern. The optional recruitment function, r, and the adoption function, a, defined in the Broadcaster pattern, are omitted in order to simplify the control algorithm.

**Figure 15:**
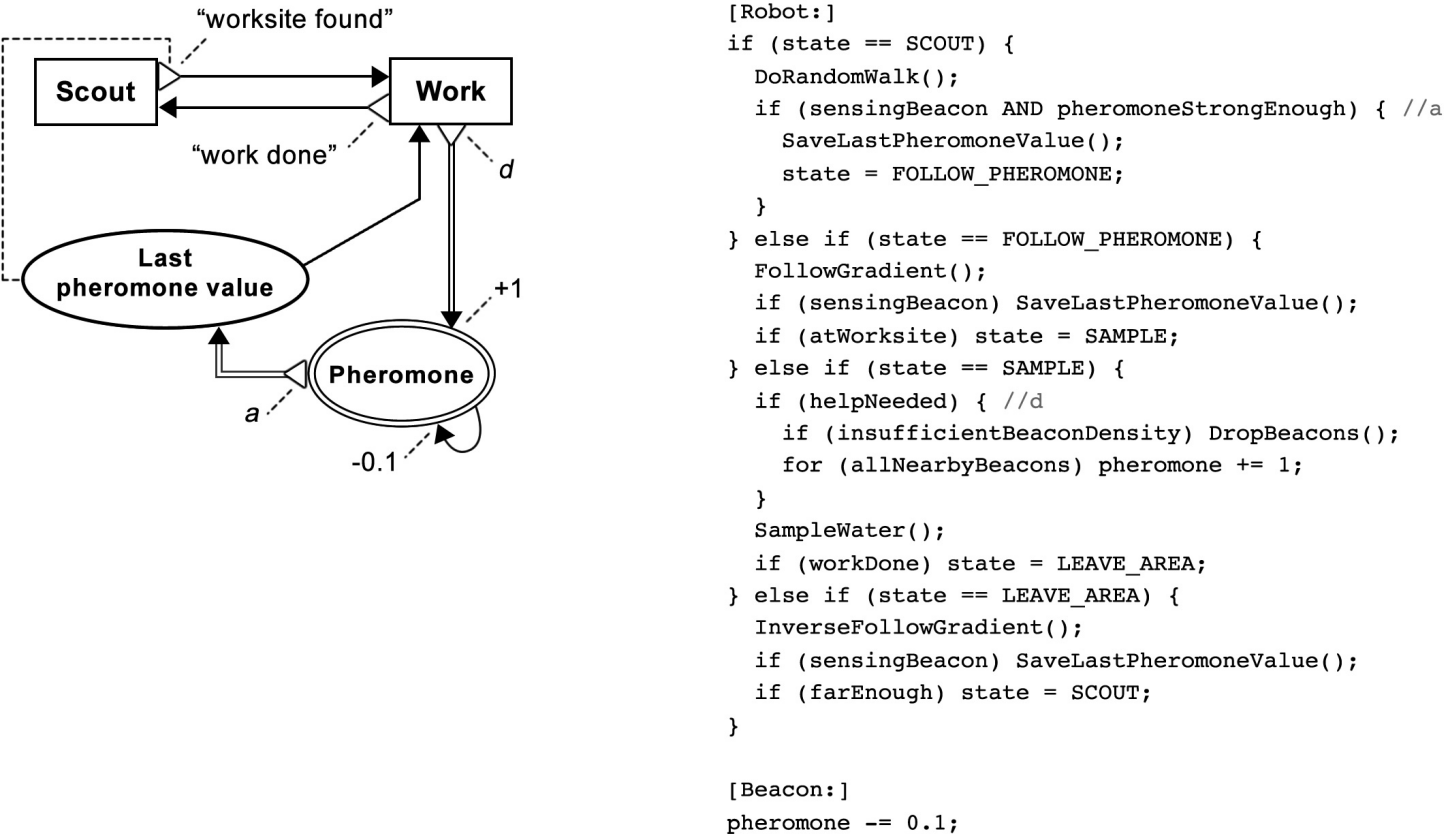
Graphical BDRML representation of the Ant-Inspired robot control algorithm, resulting from a combination of the Information Storage and Information Exchange Anywhere patterns, and update loop pseudocode for robots and beacons. Implementations of BDRML relation conditions have been indicated in the pseudocode following the *//* characters.

In the final example, autonomous submarines need to take water quality samples of contaminated areas, locations of which are initially unknown. Additionally, the range and reliability of peer-to-peer submarine communication is low, while the total area that the submarines need to search is large. In order to deal with these challenges, the Information Storage and Information Exchange Anywhere (IEA) patterns could be combined. The submarines could deposit programmable beacons around the areas of interest that would hold virtual pheromone and attract other submarines in order to decrease the mission completion time. Such “Ant-Inspired” algorithm (Figure 15) would lead to the creation of stigmergic cues, eliminating the need of the submarines to communicate with each other directly. Similar algorithms have been implemented e.g., in (Hoff et al., 2010; Hrolenok et al., 2010; Ducatelle et al., 2011).

Unlike the alternative Information Aggregation patterns, the role of the IEA pattern is to allow data to be transferred as often as necessary. Rather than causing deletion or addition of primitives, the pattern allows all primitives from the Information Storage pattern to be included after applying Rules C1-C4. On the other hand, the Information Storage pattern can redefine the relation between the “Work” and “Worksite Data Int.” primitives defined in the IEA pattern by applying Rule RDF1, where the text it write relation between these primitives is not copied, since it represents a shorter relation path. The data structures in the resulting control algorithm are renamed to “Pheromone” and “Last pheromone value”. In the state machine pseudocode implementation, the “Work” behaviour is represented by two states, “FOLLOW_PHEROMONE” and “LEAVE_AREA”. The patterns are concretised (Rule CNC1) by specifying the ways in which the *send* and *update* relations change the value of “Pheromone”.

## 6. Discussion

### 6.1. Other Swarm Design Methods

Apart from design patterns, a number of alternative methods for robot behaviour design exist. For instance, a control algorithm inspired by animal behaviour, usually that of social insects (as in e.g., Fujisawa et al., 2014; Valentini et al., 2016), or by a previously created robot control algorithm (as in e.g., Gutiérrez et al., 2010; Ducatelle et al., 2014) can be chosen by hand. Parameter values for robot control algorithms can then be selected, for example, by applying macroscopic swarm models (e.g., Reina et al., 2015; Scheidler et al., 2016; Valentini et al., 2016).

Automated design methods include, for example, on-line learning and artificial evolution. On-line learning can be used to adapt robot control parameters during swarm operation in order to respond to environmental dynamics, such as a changing number of worksites (e.g., Liu et al., 2007; Wawerla and Vaughan, 2010) or the amount of congestion (e.g., Yang et al., 2009). However, these methods are unable to deliver new control strategies in response to a significant environmental change. On the other hand, artificial evolution can be applied to create a full multi-robot control algorithm with minimal human intervention, for example by learning a neural network configuration, in an off-line (Sperati et al., 2011; Francesca et al., 2014) or an on-line (Bredeche et al., 2009) fashion. However, this technique is often not only computationally expensive and dependent on frequent communication between robots, but, more importantly, it generally delivers viable solutions relatively slowly due to a considerable amount of randomness in the evolutionary processes (Doncieux et al., 2015). Moreover, the resulting evolved robot controllers may be difficult to analyse and understand due to the their black-box nature. The last problem mostly results from using neural networks as evolved robot controllers, an approach which can be improved on by evolving combinations of pre-defined low-level robot behaviours instead (Ferrante et al., 2015).

All these methods can benefit from the knowledge that design patterns offer in the following ways. Firstly, by allowing a swarm designer to consider the properties of various building blocks of robot behaviour, design patterns can guide manual algorithm selection. Secondly, when using macroscopic or agent-based swarm models, alternative control algorithms can be constructed with the guidance of the Design Pattern Catalogue and their macro-level characteristics compared. Knowledge of suitable design patterns can also make the search space of evolutionary algorithms smaller, saving computational requirements and time. Similarly, low-level behaviour blocks based on design patterns can provide robots with alternative behaviours to switch between during on-line adaptation, as was previously suggested in (Hernández et al., 2013; Ferrante et al., 2015).

From a different perspective, making design pattern creation one of the goals of experimentation and analysis in swarm robotics can inspire working towards a deeper understanding of collective intelligence by studying the mechanisms that play a role in the micro-macro link [e.g., as in (Reina et al., 2015)]. Many authors have argued that design and analysis approaches that could capture general principles of how emergent collective behaviour comes about are crucial to the advancement of the field (e.g., Parunak and Brueckner, 2004; Serugendo et al., 2006; Winfield, 2009b; Brambilla et al., 2014). Design patterns can represent a way of making this type of understanding explicit.

### 6.2. Other Design Pattern Work

It has been previously suggested that design patterns for swarm robotics should describe multiple levels of behaviour. For example, “local-level” or “basic” patterns have been used to describe how robots interact, while “global-level” or “composed” patterns described swarm-level behaviour, such as “Labour division” (Nagpal, 2004; Gardelli et al., 2007; Fernandez-Marquez et al., 2013). On the contrary, all design patterns presented here describe the same level of robot behaviour, equivalent to “local-level primitives” (Nagpal, 2004) or “basic design patterns” (Fernandez-Marquez et al., 2013). Control algorithms then result from combining the design patterns and are thus their realisations, assembled to fit a particular mission. The control algorithms are equivalent to “global-level primitives” (Nagpal, 2004) or “high-level patterns” (Fernandez-Marquez et al., 2013) found in the literature.

The focus on robot behaviours and data structures that they utilise and manipulate, and on describing small sub-components of robot control algorithms, is an important aspect of the design patterns presented here. A similar approach has been taken for distributed communication design patterns, where biologically-inspired local communication strategies were defined and combined into control algorithms for specific applications (Babaoglu et al., 2006). In our view, description of “global-level” swarm behaviour would be a re-description of multiple local-level patterns and of their dependencies on and interactions with each other. Swarm behaviour can be complex, and in some cases emergent, which makes it important to identify minimal sets of behaviours that can be thoroughly analysed and their ability to achieve or avoid outcomes at the collective level described. In a well-specified framework, that includes a formal behaviour definition, such as the one offered by BDRML, a list of feedback loops, forces and pattern consequences on swarm-level behaviour, as well as unabmiguos application rules, a separate specification of control algorithms that may be created from these patterns should not be necessary. For the design patterns presented in this paper, a demonstration of how different patterns can be considered, combined and implemented has been presented in Section 5.

The second aspect in which the design patterns presented here differ from those in the swarm robotics literature is the introduction of categorisation, which follows the methodology of object-oriented design patterns (Gamma et al., 1994) and of design patterns for distributed software algorithms (Aridor and Lange, 1998). The purpose of categorising design patterns is, first, to make it easier to specify their roles, and in turn to identify which patterns can be combined together. In most cases, only patterns from different categories will be combined as they are responsible for different aspects of robot behaviour. Secondly, a design pattern category may suggests which optional Application Rules are appropriate to use when creating a control algorithm, as is, for example, the case for Rule EXT1 (Section 3.2).

### 6.3. Current Issues and Future Work

Despite their advantages, the swarm design patterns presented here currently have a number of issues that need addressing. The design patterns need to be tested in a larger number of scenarios, which will facilitate their better specification. For example, the Broadcaster pattern description suggests that the pattern should be used when “the combination of robot and worksite density makes it difficult for robots to discover worksites.”. Is it possible to quantify this “difficulty”? Is there a specific threshold for worksite and robot density that clearly distinguishes environments where this pattern is and is not suitable? Or, more generally, is there a way of matching the parameters of a design pattern to parameters of the swarm task? While the ICR framework (Pitonakova et al., 2018) or other analytical approaches (Reina et al., 2015) can be used to analyse the forces that play a role in the micro-macro link, the fact that design patterns presented here are modular and can be combined together in different ways makes it difficult to exhaust all possibilities in which they can manifest themselves in swarm-level behaviour. It is therefore possible that we will never be able to identify, with a complete certainty, the most suitable set of design patterns for a given mission simply by browsing the Design Pattern Catalogue. However, more experiments can be performed with each design pattern, for example with different robot hardware and in different scenarios, to make the pattern specifications more detailed. In particular, the following challenges could be addressed:

- Characterising the interaction of the Information Exchange Anywhere pattern with various Information Transmitter patterns. A larger set of consequences for the Information Exchange Anywhere pattern needs to be specified.
- Characterising in more detail the effect of communication range in the Broadcaster pattern and of detection range in the Information Storage pattern
- Characterising in more detail the effect of proximity threshold in the Information Exchange near Worksites pattern
- Characterising the effect of swarm size and robot density on all design pattern properties

On the other hand, it is also important to note that, by offering a list of forces and consequences, a design pattern can identify situations in which it *should not* be used. For example, the Broadcaster pattern only works when sufficient communication range between robots is available. It could be combined with the Information Exchange Centre in order to alleviate this problem, but only during central-place foraging.

Rather than thinking about design patterns as branches of a decision tree, that asks us questions about the robots and the environment and eventually leads us to one suitable solution, we should think about the Design Pattern Catalogue as a source of alternatives that a robot designer, or an automated design algorithm, can consider. A similar approach has been taken in the case of object-oriented (Gamma et al., 1994) and distributed computing (Babaoglu et al., 2006) design patterns, where it is up to the developer to decide which patterns to use and how to implement them together based on the context of the specific application that is being developed. In any case, having design guidelines at hand may be a better alternative to creating an entire robot control algorithm based on arbitrary decisions.

Apart from providing better specifications for the existing patterns, the Design Pattern Catalogue also needs to be extended in a number of ways. Firstly, when it comes to foraging, design patterns for more types of robot behaviours could be created, for example those for scouting the environment, robot localisation, updating of information about worksites, etc. This could be achieved by performing targeted comparative experiments with different parts of robot behaviours, similarly as in the studies that inspired creation of the design patterns presented here (e.g., Gutiérrez et al., 2010; Wawerla and Vaughan, 2010; Sarker and Dahl, 2011; Pitonakova et al., 2014, 2016a, Pitonakova et al., 2018). Secondly, the Design Pattern Catalogue should consider a larger number of swarm tasks, such as dispersion, aggregation or collective construction. It would be interesting to find out whether the foraging patterns presented here would be applicable to other swarm tasks and whether their descriptions could be improved by studying them in new contexts. It is reasonable to assume that some patterns could be generalised enough to be applicable across different tasks. For example, the Broadcaster pattern has been used in multi-robot flocking (Vasarhelyi et al., 2014) and aggregation (Couceiro et al., 2012), to allow robots to share information about their positions, intentions and the landscape of the environment. During a different, construction task, the “Work” behaviour of the Broadcaster pattern could be interpreted as “Build” and “Worksite” would be equivalent to a “Building site”.

Further extensions of the Design Pattern Catalogue could include new patterns for heterogeneous robot swarms and swarms that work alongside humans or report data to users. In these cases, entities that have different types of behaviour would work with each other, making it more difficult to identify consequences of patterns due to increased system-level complexity. Therefore, descriptions of design pattern consequences would need to include specific conditions under which they occur. For example, a behaviour might incur different costs when a robot executing it works alongside humans rather than within a homogeneous swarm.

Environments with more complex dynamics also need to be explored. For example, it is reasonable to assume that some robot missions would involve worksites with different priorities, or worksites that could only be serviced by a subset of robots. Tasks that require strong cooperation between robots, such as collective transport, are also an interesting challenge. In other missions, the ability of robots to perform work or to navigate the environment may change over time, for example due to varying weather or city traffic conditions. Understanding the impact of environmental dynamics on the ability of robots to obtain, share and utilise information is vital for both refining and extending the Design Pattern Catalogue.

Finally, the rules for applying design patterns may need to be refined and new rules may need to be added when mode design patterns and application case studies are available. For example, it is possible that future design patterns could be used to restrict information processing routines by shortening relation paths between pattern primitives. Some Application Rules may only be applicable to a certain design pattern category, for example, as is the case for Rule EXT1.

In a more general sense, it is interesting to think about how the concept of “interfaces” from object-oriented software engineering (Gamma et al., 1994) extends to robot swarm algorithms. Can “interfaces” between swarm sub-algorithms ever be well-defined, given the emergent nature of the systems that they result in when combined together? While the divide-and-conquer approach that design patterns suggest may restrict us to a more traditional engineering part of the design space, is this the trade-off that has to be paid for obtaining a well-understood and well-defined design methodology for complex systems such as robot swarms?

As the previous few paragraphs suggest, the work needed to extend and maintain a design pattern catalogue for robot swarms is extensive. Therefore, it is our goal to make the Design Pattern Catalogue presented here publicly available and extendable^[6]^. BDRML will also need to be refined and extended, so that it is able to accommodate a broader range of patterns and represent various heterogeneous entities that share information with each other. Furthermore, implementation examples and code libraries could be added in order to facilitate the usage of design patterns for specific robots and applications. Object-oriented design patterns nowadays come with implementation examples in various programming languages (Shalloway and Trott, 2005), which makes them easier to understand and apply.

## 7. Conclusion

Robot swarms are a promising technology that could transform the way in which we manage logistics, transportation and agriculture, how we take care of the environment, and how we explore and colonise new planets. One of the greatest challenges of using this technology is that it is not obvious how we can relate robot behaviour, programmable by software developers, to a desired swarm-level outcome. Design patterns can serve as guidelines to algorithm design by capturing properties and effects of robot behaviours on swarm performance. Secondly, they can be useful for checking whether a particular algorithm is suitable given particular swarm task characteristics and availability of hardware, and for providing alternative solutions that can be considered. Thirdly, design patterns can guide automated design methods, such as adaptive algorithms and artificial evolution, as well as reduce their computational requirements.

This paper presented information exchange design patterns for robot swarm foraging. The patterns were modular in nature - they represented a particular aspect of robot behaviour, for example a location at which information should be exchanged between robots, and they could be combined into robot control algorithms. An important objective of each pattern was to describe the feedback loops and forces that affected the pattern’s effectiveness, as well as the pattern’s consequences on swarm-level behaviour. This was achieved by applying the Information-Cost-Reward framework for swarm behaviour analysis (Pitonakova et al., 2018) and for interpreting analysis results from studies found in the literature. The behaviour description and combination of the patterns were facilitated by the Behaviour-Data-Relations Modelling Language (Pitonakova et al., 2017), in which relevant robot behaviours and data structures, as well their relationships, can be expressed explicitly. The patterns were validated by demonstrating how they improved the performance of foraging e-puck swarms and how they could guide algorithm design in other scenarios. The ability of the design pattern specification method presented here to identify distinguishing features of robot behaviour and their impact on swarm performance in a wide range of swarm implementations and experimental scenarios was demonstrated by successfully applying it to reason about and to organise robot behaviours implemented by other authors.

## Author Contributions

LP was responsible for the conception of the work, literature review, creation of the ICR framework, BDRML language and the design patterns, implementation and execution of simulations, robot experiments and data analysis software, and drafting and finalising this manuscript. RC and SB contributed by critically revising the work, in particular the ICR framework, the BDRML language and the design patterns (SB) and the robot experiments (RC).

## Conflict of Interest Statement

The authors declare that the research was conducted in the absence of any commercial or financial relationships that could be construed as a potential conflict of interest.

## Footnotes

1. http://bit.ly/2HwdnlT

2. This requirement is similar to the requirement of object-oriented design patterns for programming to an interface rather than to an implementation, whichleads to reusability and minimal implementation dependencies (Gamma et al., 1994, p. 30).

3. An “or” condition defines additional situations in which a relation is applicable. In the most extreme case of the condition being empty, “or false” is aredundant expressions and can therefore be omitted.

4. The “always” condition always returns “true” and it should therefore be combined with other conditions using the “and” operator, ensuring that the otherconditions are satisfied according to their specifications. Since “and true” is a redundant expression, the “always” condition can be deleted.

5. “Worksite data ext.” would be included if, for example, the Information Storage and Information Exchange Centre patterns were combined, since Information Storage is an Information Transmitter pattern that includes a copy relation between “Worksite data ext.” and “Worksite data int.”. Such an algorithm has, for example, been implemented in (Alers et al., 2011).

6. http://designing-robot-swarms.lenkaspace.net
